# A Sliced Inverse Regression (SIR) Decoding the Forelimb Movement from Neuronal Spikes in the Rat Motor Cortex

**DOI:** 10.3389/fnins.2016.00556

**Published:** 2016-12-09

**Authors:** Shih-Hung Yang, You-Yin Chen, Sheng-Huang Lin, Lun-De Liao, Henry Horng-Shing Lu, Ching-Fu Wang, Po-Chuan Chen, Yu-Chun Lo, Thanh Dat Phan, Hsiang-Ya Chao, Hui-Ching Lin, Hsin-Yi Lai, Wei-Chen Huang

**Affiliations:** ^1^Department of Mechanical and Computer Aided Engineering, Feng Chia UniversityTaichung, Taiwan; ^2^Department of Biomedical Engineering, National Yang Ming UniversityTaipei, Taiwan; ^3^Institute of Biomedical Engineering, College of Medicine, National Taiwan UniversityTaipei, Taiwan; ^4^Department of Neurology, Tzu Chi General HospitalTzu Chi University, Hualien, Taiwan; ^5^Institute of Biomedical Engineering and Nanomedicine, National Health Research InstitutesZhunan Township, Taiwan; ^6^Singapore Institute for Neurotechnology, National University of SingaporeSingapore, Singapore; ^7^Institute of Statistics, National Chiao Tung UniversityHsinchu, Taiwan; ^8^The Ph.D. Program for Neural Regenerative Medicine, College of Medical Science and Technology, Taipei Medical UniversityTaipei, Taiwan; ^9^Department of Electrical Engineering, National Taiwan UniversityTaipei, Taiwan; ^10^Department and Institute of Physiology, School of Medicine, National Yang Ming UniversityTaipei, Taiwan; ^11^Interdisciplinary Institute of Neuroscience and Technology, Qiushi Academy for Advanced Studies, Zhejiang UniversityHangzhou, China; ^12^Department of Materials Science and Engineering, Carnegie Mellon UniversityPittsburgh, PA, USA

**Keywords:** sliced inverse regression (SIR), neural decoding, forelimb movement prediction, neural networks (NN), principle component analysis (PCA)

## Abstract

Several neural decoding algorithms have successfully converted brain signals into commands to control a computer cursor and prosthetic devices. A majority of decoding methods, such as population vector algorithms (PVA), optimal linear estimators (OLE), and neural networks (NN), are effective in predicting movement kinematics, including movement direction, speed and trajectory but usually require a large number of neurons to achieve desirable performance. This study proposed a novel decoding algorithm even with signals obtained from a smaller numbers of neurons. We adopted sliced inverse regression (SIR) to predict forelimb movement from single-unit activities recorded in the rat primary motor (M1) cortex in a water-reward lever-pressing task. SIR performed weighted principal component analysis (PCA) to achieve effective dimension reduction for nonlinear regression. To demonstrate the decoding performance, SIR was compared to PVA, OLE, and NN. Furthermore, PCA and sequential feature selection (SFS) which are popular feature selection techniques were implemented for comparison of feature selection effectiveness. Among SIR, PVA, OLE, PCA, SFS, and NN decoding methods, the trajectories predicted by SIR (with a root mean square error, RMSE, of 8.47 ± 1.32 mm) was closer to the actual trajectories compared with those predicted by PVA (30.41 ± 11.73 mm), OLE (20.17 ± 6.43 mm), PCA (19.13 ± 0.75 mm), SFS (22.75 ± 2.01 mm), and NN (16.75 ± 2.02 mm). The superiority of SIR was most obvious when the sample size of neurons was small. We concluded that SIR sorted the input data to obtain the effective transform matrices for movement prediction, making it a robust decoding method for conditions with sparse neuronal information.

## Introduction

In order to improve daily life activities for paralyzed patients, the establishment of a non-muscular communication interface between brain neurons and machines has rapidly developed over the last two decades (Schwartz, 1993, 1994; Donoghue, 2002; Schwartz et al., 2006; Velliste et al., 2008). With assistance from stable generated brain-derived control signals incorporated with prosthetic devices and motor functions, paralyzed patients now possibly regain their ability to move a computer cursor (Kennedy et al., 2000; Hochberg et al., 2006; Gilja et al., 2015), control an anthropomorphic prosthetic arm (Wodlinger et al., 2015), and drive a prosthetic device (Hochberg et al., 2012; Collinger et al., 2013) through a brain-machine interface (BMI). One important challenge to BMI is how to design an appropriate neural decoder (Pohlmeyer et al., 2014). To address the challenge, previous studies have carefully utilized training paradigms that have been designed for a BMI decoder and controller. For brain-derived control signals, neural decoding is an indispensable technique that translates neuronal activities to physical states, such as the position of a foraging rat (Brown et al., 1998), arm movement (Ashe and Georgopoulos, 1994), movement speed (Moran and Schwartz, 1999), hand position (Paninski et al., 2004), and joint angular velocity (Reina et al., 2001).

A population vector algorithm (PVA), one method for decoding motor cortical activity, assumed that a neuron's firing rate is related to the velocity vector of movement. PVA categorizes each neuron's contribution into directional and distance information of the movement by a directional tuning function under uniform variance conditions (Georgopoulos et al., 1988). A previous study showed that PVA decoding could expose the visuomotor coordinate transformations between visual and motor information by processing masses of neuronal activities recorded from relative brain regions (Takeda and Funahashi, 2004; Watanabe et al., 2009). PVA presented superior performance in predicting hand path throughout reaching tasks (Schwartz, 1994). However, a uniformity constraint is usually not the case for real experiments, and the equality of the tuning function is variable because of the small amount of unit recordings in realistic applications (Schwartz et al., 2001). To compensate for the non-uniform preferred directions in the population of recorded neurons, an optimal linear estimator (OLE) was proposed to define the preferred direction of each neuron using the center of mass of the tuning function (Salinas and Abbott, 1994). Requiring large numbers of neurons with a temporal solution of 10–100 ms, PVA and OLE studies successfully predicted the kinematic parameters of a primate arm movement (Schwartz et al., 2001; Takeda and Funahashi, 2004; Watanabe et al., 2009).

A Bayesian decoder, a probabilistic decoding technique, could achieve accurate offline trajectory reconstructions by combing simple trajectory models (Yu et al., 2007). However, off-line reconstruction may not be suitable for online prosthesis control because the essential features of a real prosthesis are not acquired, and the system dynamics may vary because the user is in a closed loop. Furthermore, offline and online approaches resulted in different parameter choices for decoding algorithms (Cunningham et al., 2011). Therefore, the neural decoders and the motor prosthesis must be tested online even though online control experiments are more expensive both in terms of physical resources and time (Gilja et al., 2011). A recursive Bayesian decoder, i.e., a Kalman filter, was developed to decode the neural data recorded in the monkey motor and premotor cortex in response to goal-directed reaching movements (Shenoy and Carmena, 2014). It yielded high decoding performance and accurate trajectory prediction when the probability modeling assumptions were satisfied. For online purposes, a modified Kalman filter that transforms the acquired neural signals into a controller input was further designed for online cursor-control tasks and resulted in high performance in rhesus monkeys (Gilja et al., 2012). To adapt decoders to the dynamics of a prosthetic device and its environment, a likelihood gradient ascent and a self-recalibrating classifier were proposed to update decoder parameters during closed-loop BMI operation and normal use (Dangi et al., 2013; Bishop et al., 2014). Additionally, neural networks developed from probabilistic aspects were designed in an online setting (Sussillo et al., 2012) and in a real-time setting (Dethier et al., 2013).

A selection of cortical neurons, instead of all available neurons, used in the encoding process could improve the control performance of the neuroprosthetic system, such as robotic arms (Wahnoun et al., 2006). However, neural coding mechanisms evolve with time, individual experience, and the learning process (Nicolelis, 2001), i.e., the contribution from individual neurons may vary considerably from day to day (Carmena et al., 2003). It has been observed that neuronal activity for monkey was not as stable from day to day (Sadtler et al., 2014). The decoding algorithms which require previous day's observation of neural activity, such as PVA and OLE, may be affected by neuron's stability. For this reason, the selection of cortical neurons becomes an essential issue for the decoding processes, especially after continuous practice and learning. Furthermore, long-term inflammatory responses lead to a gradual decrease of recording quality and the eventual breakdown of the electrode's recording ability (Polikov et al., 2005; Schwartz et al., 2006). Losing neural signals over time will result in chronic coding failure. Thus, a decoder that has the capability to process recorded signals from a small number of neurons will become more important.

Sliced inverse regression (SIR) is a data-analytic tool that can effectively perform nonlinear regression based on a small number of inputs (Li, 1991). It divides the range of output variable into several intervals and partitions the input data into several slices according to the corresponding output value. Each slice consists of data with a similar contribution to output estimation. SIR then applies a weighted principal component analysis (PCA) to theses slice means of data to find effective dimension reduction directions for general and flexible setups. Each slice gains weight according to its contribution to output estimation. With a simple inverse regression model, SIR requires a low computational cost and retains reliably stable subspaces to extract primary information from noisy data with effective dimension reduction directions. Because of the good performance in dimension reduction and data de-noising, SIR has been widely applied to data-intensive marketing environments (Naik et al., 2000), data classification (Dai et al., 2006; Wu, 2008), and medical images (Wu and Lu, 2007; Lu, 2008; Tu et al., 2015).

It has been known that the number of recorded neurons in rodents is less than primates in BMI applications. Owing to the abilities of processing small number of input variables and slicing data for inverse regression model, SIR was considered as a neural decoding algorithm to provide realistic behavior interpretation for brain-derived control tasks in this study. A surrogate driving task with lever-pressing was designed for evaluating the efficacy of the proposed decoding algorithm in predicting motor functions of rats. The signals of lever-pressing and the spike trains related to the intended forelimb movement trajectories were simultaneously acquired during the task. This study adopts SIR to predict intended forelimb movement trajectories according to the recorded neurons from primary motor (M1) cortex. We presented experimental validation of the proposed decoding system using the recorded neurons to predict forelimb movement. We demonstrated that the proposed SIR decoding algorithm can not only extract primary features from the small number of neurons but perform more accurate prediction of intended forelimb movement trajectories than PVA and OLE which usually require hundreds of neurons in primates.

## Materials and methods

### Animal preparation

Four male Wistar rats weighing 250–300 g (BioLASCO Taiwan Co., Ltd., Taiwan) were used in this study. All rats were individually housed in a 12 h light-dark cycle (light from 7.00 to 19.00 h) at room temperature (22 ± 1°C) with access to food and water *ad libitum* in the experimental animal center of National Yang Ming University. All experiments were conducted according to standards established in the Guide for the Care and Use of Laboratory Animals, which has been approved by the Institutional Animal Care and Use Committee at the National Yang Ming University.

### Animal training and behavioral tasks

**Figure 1:**
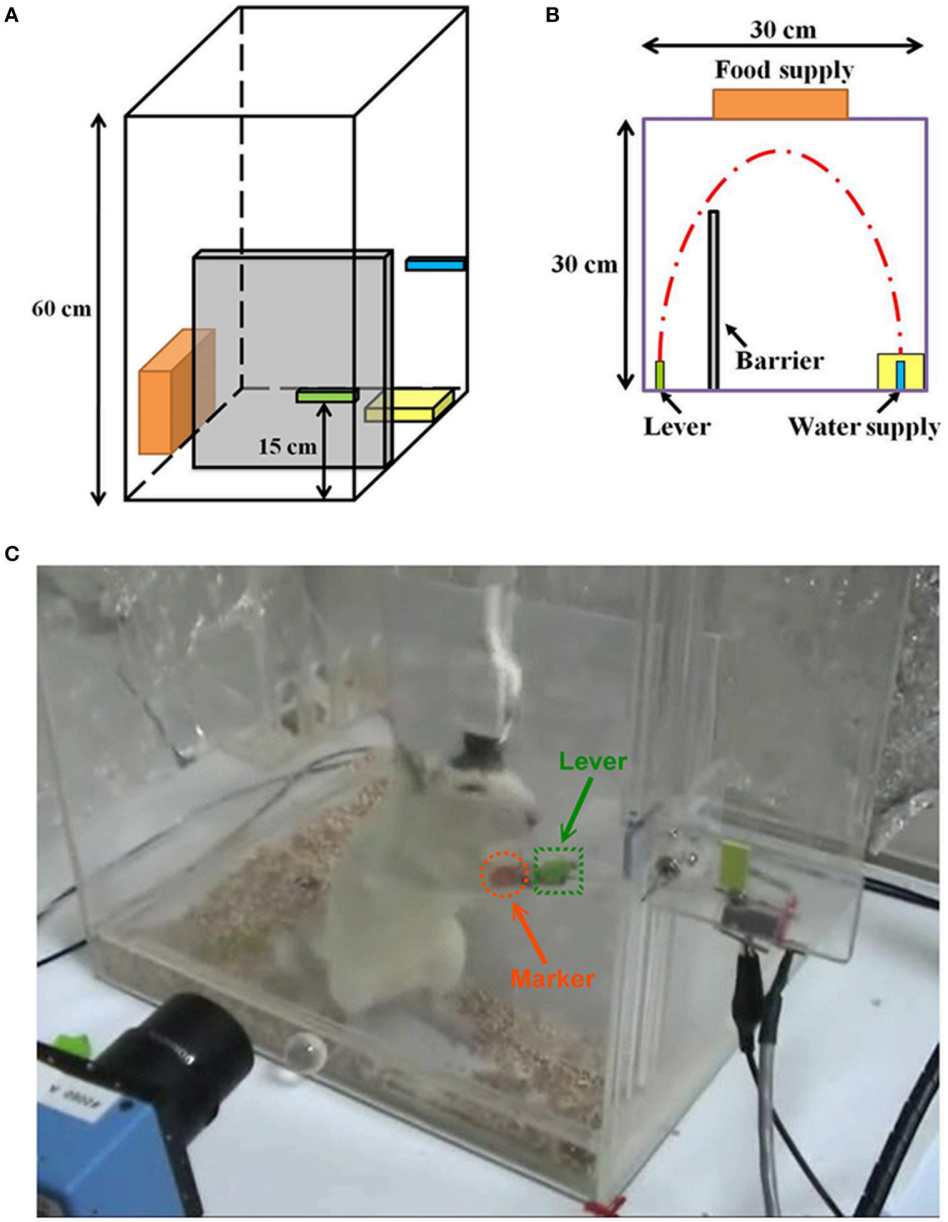
**Experimental setup and protocol**. A perspective drawing **(A)** and vertical view **(B)** of the Plexiglas testing box. A lever is on the left side of the barrier and a water supply is on the right side. **(C)** A rat is using his right forelimb to press the lever to obtain a water reward. Simultaneously, his forelimb movement trajectory is videotaped by a camcorder approximately 25 cm away from the box, and the neuronal activities are recorded by the implanted electrode.

The rats were trained to use their right forelimb to press a lever to obtain the water reward for a week before electrode implantation, as shown in Figure 1. The rat was placed in the lab-designed Plexiglas testing box with a 15-cm tall lever above the floor on the left side (Figure 1A) and a water-feeder with a flow rate of 1 ml/time on the right side as shown in Figure 1B (Lin et al., 2016). Figure 1C shows the experimental setup where a rat was pressing a lever for the water reward. Before achieving the successful lever-pressing training, all rats underwent water deprivation for 8 h per day. In this study, we have defined the criterion for the successful training was to consecutively repeat the lever-pressing and water-drinking for five times during daily 5-h sessions (9:00–14:00), for 4 days at the most. Once the rats learned the association, they always kept the skilled concept (Lin et al., 2016).

### Surgery and electrophysiological mapping

Animals were anesthetized with pentobarbital (50 mg/Kg, i.p.) and were placed on a stereotaxic apparatus (Model 900, Kopf Instruments, Tujunga, CA, USA). A 16-channel stainless microwire electrode array (diameter of 0.002 ft., California Fine Wire Co., Ltd, Grover Beach, CA, USA) was inserted vertically and was implanted into layer V of the M1 cortex (2–4 mm anterior and 2–4 mm left-lateral to bregma; 1.7 mm ventral to the cortical surface) by referring to a previous work. Here, a standard intracortical microstimulation (ICMS) technique was conducted to deduce maps of rat forelimb movement representations in the M1 cortex, which could help assess the functional integrity of M1 cortex and activate pyramidal cell fibers. ICMS delivered a 40 ms stimulus train with 0.2 ms square-wave monophasic cathodal pulses at 350 Hz to the electrodes (impedance: 200–400 kΩ at 1 kHz) by an isolated pulse stimulator at a rate of 1/s (Model 2100, A-M Systems Inc., Sequim, WA, USA). Because the intensity of the stimulating current depends on the distance between the neuron and the stimulating electrode, the threshold current intensity can be estimated by a strength-distance relationship as follows:

(1) $$\begin{array}{l} I=kr^{2}+I_{m} \end{array}$$

where *k* = 1292μA/mm^2^ for direct activation, *r* is the distance, and *I*_*m*_ = 1μA. An implantation location of the electrode site was defined as valid when the rat forelimb was activated by ICMS with a current intensity less than 60μA. Then, a stainless steel screw was secured to the skull over the cerebellum as a reference electrode. Finally, the microwire electrode array was secured in the skull using dental acrylic (Type 1 Class 1, Hygenic Corp., Akron, OH, USA) and was covered with a small amount of 2% agar. For a better recovery, all rats were given an analgesia (Buprenorphine/Buprenex, 0.05 mg/kg s.c.; Reckitt Benckiset Healthcare Ltd, Hull, UK) every 8–12 h for 3 days and antibiotic treatment (Ampicillin, 100 mg/kg s.c. twice daily; Bristol Myers Squibb, New York, NY, USA) for 7 days after surgery. Following a 1-week post-surgery recovery period, all implanted rats received the behavioral task to use their forelimb to press a lever for water reward. The forelimb movements in the rat were captured by a charge coupled device (CCD) camera (DFK 21F04, Imaging Source, Bremen, Germany) and the neuronal signals were recorded by a Multi-channel Acquisition Processor (MAP, Plexon Inc., Dallas, TX, USA) through a 16-channel stainless microwire electrode array implanted in the rat M1 cortex. The detailed data recordings for forelimb movement and neuronal signals are described in the Supplementary Note 1.

### Trajectory prediction model

This study assumed that lever-pressing forelimb movement, which was considered to be a stereotyped movement, was performed at a nearly constant distance from the CCD in each trial, i.e., the distance did not vary dramatically. The recorded trajectory might consist of major forelimb movement and minor whole body movement which led to the coupling mechanism of two-dimensional forelimb movement (see Supplementary Note 1). The coupling mechanism resulted in a nonlinear relationship between neural activity and forelimb movement, and thus caused general linear regression to fail at forelimb movement prediction. SIR performs as a nonlinear regression since it can recover the most severe nonlinearity of the data by estimating effective dimensional reduction (*e.d.r*.) space (Li, 1991). Therefore, SIR was adopted to predict the forelimb movement according to the neural activity in this study.

The two-dimensional trajectory movement vectors *v*_*x*_ and *v*_*y*_ in Cartesian coordinates are transformed into polar coordinates as follows:

(2) $$\begin{array}{c} v_{r}=\sqrt{v_{x}^{2}+v_{y}^{2}} \end{array}$$

(3) $$\begin{array}{c} v_{\theta }=\tan^{-1}\frac{v_{y}}{v_{x}} \end{array}$$

where *v*_*r*_ and *v*_θ_ are the magnitude and direction, respectively.

The movement response *g* is assumed to be given by a deterministic function *f* with additive noise ε, so that

(4) $$\begin{array}{l} g=f\left(\beta_{1}z,\;\ldots ,\beta_{K}z,\varepsilon \right) \end{array}$$

where *g* is *v*_*r*_ or *v*_θ_, and *z* is firing rate data in **R**^*p*^. Here, β's are unknown linearly independent projection row vectors, and *K* is the sufficient number of β's. The *p*-dimensional variable *z* is projected onto the *K*-dimensional space by functional relationship *f*, where *p* ≥ *K*. The combinations of β's are called the *e.d.r*. direction and the linear space produced by the β's are called *e.d.r*. space. The present study assumes the movement response *g* was predictable from *K* projected variables. To train the functional relationship *f*, a set of training data consisting of *N* training samples was prepared. According to the model assumptions, the centered inverse regression curve *E*(*z*|*g*) − *E*(*z*) is included in the linear subspace, which is spanned by β_*k*_Σ_*zz*_(*k* = 1, …, *K*), where Σ_*zz*_ represents the covariance matrix of *z*. SIR sorts and divides the whole data *z* into *H* intervals (slices) according to the *g* value. Each slice has almost equally number of observations. SIR then performs an eigenvalue decomposition of the weighted covariance matrix Σ_*E*(*z*|*g*)_ with respect to Σ_*zz*_. The weighted covariance matrix Σ_*E*(*z*|*g*)_ is constructed as:

(5) $$\Sigma_{E\left(z|g\text{​}\right)}=\frac{\sum_{h\;=\;1}^{H}m_{h}\text{​}\left({\bar{z}}_{h}-\bar{z}\right){\left({\bar{z}}_{h}-\bar{z}\right)}^{\prime }}{\left(N-1\right)}$$

where *m*_*h*_ denotes the size of each slice, $\bar{z}$ is the sample mean of *z*, and ${\bar{z}}_{h}$ is the sample mean of the *h*th slice. The *e.d.r*. directions could be estimated by solving the generalized eigen-problem:

(6) $$\Sigma_{E\left(z|g\text{​}\right)}\beta_{j}=\lambda_{j}\Sigma_{zz}\beta_{j}$$

where *j* = 1, …, *p* and λ_1_ ≥ λ_2_ ≥ ⋯ ≥ λ_*p*_. Then, *z* was further projected onto the *e.d.r*. space by the first *K e.d.r*. directions as follows:

(7) $$w=\left[\beta_{1}z,\;\ldots ,\beta_{K}z\right]$$

Then, a linear combination of *w* was performed to predict the forelimb movement. Although a linear combination approach was adopted, SIR was considered as a nonlinear regression since there is no linearity constraint on the prediction rules. Note that the user-specified parameters of SIR are only the number of slices *H* and the number of components *K*. It has been known that SIR can provide root *n* consistent estimates regardless of the choice of *H*. A previous study has demonstrated that the performance of SIR is less sensitive to the selection of *H* when *H* was set 5, 10, and 20 (Li, 1991). Furthermore, it has been found that the first component (*K* = 1) is close to the *e.d.r*. space. Therefore, *H* and *K* were set to 10 and one, respectively, in this study.

### Time-lags and temporal orders

In fact, the physical relationship between the neuronal signal and the forelimb movement may imply time-lags in the neuronal signal. Previous work indicates that a model that assumes that all cells exhibit the same time-lags is computationally simple (Wu et al., 2004). Then, the optimal time-lags could be found with an empirical setting for further improvement of the decoding task. A number of time-lags (0–5 time bins, at levels corresponding from 33 to 165 ms) were evaluated for trajectory prediction by SIR.

In addition to the time-lags, the temporal order of the input is another interesting factor for the decoding issue. The information at the *n*^th^ time bin may have a relationship with that at the (*n* − 1)^th^ time bin. Hence, both current and previous neuronal activities are important and are considered as the inputs for prediction. Therefore, a tapped delay line model of neuronal activities is adopted in this study where a third-order model would consider the *n*^th^, (*n* − 1)^th^, and (*n* − 2)^th^ time bins as the input.

### Performance evaluation and statistical analysis

This study computed the root mean square error (RMSE) between true and predicted forelimb movements from movement start to endpoint in order to examine the performance of proposed decoding algorithm (Srinivasan and da Silva, 2011). The experimental trails were randomly split 70/30% into training and testing sets for each rat. Therefore, the performance of the proposed decoding algorithm on the testing set could be evaluated on each rat individually. A 10-fold cross validation was applied to avoid capitalization on chance (Efron and Tibshirani, 1994).

For statistical analysis, the predicted performance (RMSE) from 4 testing sets (145 trails; rat 74: 24 trials, rat 102: 18 trails, rat 106: 78 trails and rat 129: 25 trails) were represented as the mean ± standard error of mean (SEM). Two-way ANOVA was calculated using effects of time-lag (bin number 0, 1, 2, 3, 4, and 5) and temporal order (1-oder, 2-order, and 3-oder of time bins) as the independent variables in order to determine if there were any differences in the decoding ability based on which parameters was employed. *Post-hoc* comparisons were conducted using a Tukey HSD *post-hoc* test and the significance level was corrected to ^*^P < 0.002 using a Bonferroni correction for the comparison of six time-lags and three temporal order. MATLAB (MathWorks, Natick, MA, USA) was used for all statistical analyses.

## Results

To evaluate the decoding performance of the proposed algorithm for trajectory prediction, the majority of decoding methods, PVA, OLE, and NN, were implemented for comparison. Furthermore, two popular feature selection techniques, PCA (Wold et al., 1987) and sequential feature selection (SFS) (Aha and Bankert, 1996), were implemented for comparison of feature selection effectiveness. Although PCA was developed as dimension reduction of feature space, it could be considered as a way to select features from principle components. Then a linear regression approach was adopted to perform regression whose inputs were the features provided by PCA and outputs were forelimb movements. The number of principal components was selected according to the variance of the reconstruction error (Valle et al., 1999). The same regression procedure was applied to SFS. A set of time-lags (0–5 time-lags) was carried out to observe the effect of different delays between the neuronal activity and the forelimb movement in each method. Furthermore, a set of experiments was conducted to evaluate the effect of various temporal orders (1–3 temporal orders) used in each decoding method. The experimental data were recorded from four rats where the number of trials and number of neurons per trial for each rat are shown in Table 1. The average number of successful trials was 45.9 ± 4.3, and the average number of recorded neurons was 21.2 ± 2.6 units. The period of each trial was 0.4–0.7 s before lever-pressing and 0.2–0.4 s after the lever-pressing.

**Table 1:** **Experimental data characteristics**.

**Subject**	**Number of trials**	**Number of neurons per trial**
Rat 74	80	18±3.1
Rat 102	60	11.1±1.5
Rat 106	263	32.9±5.2
Rat 129	83	24.9±5.4

### Neuronal signal pattern during behavior task

**Figure 2:**
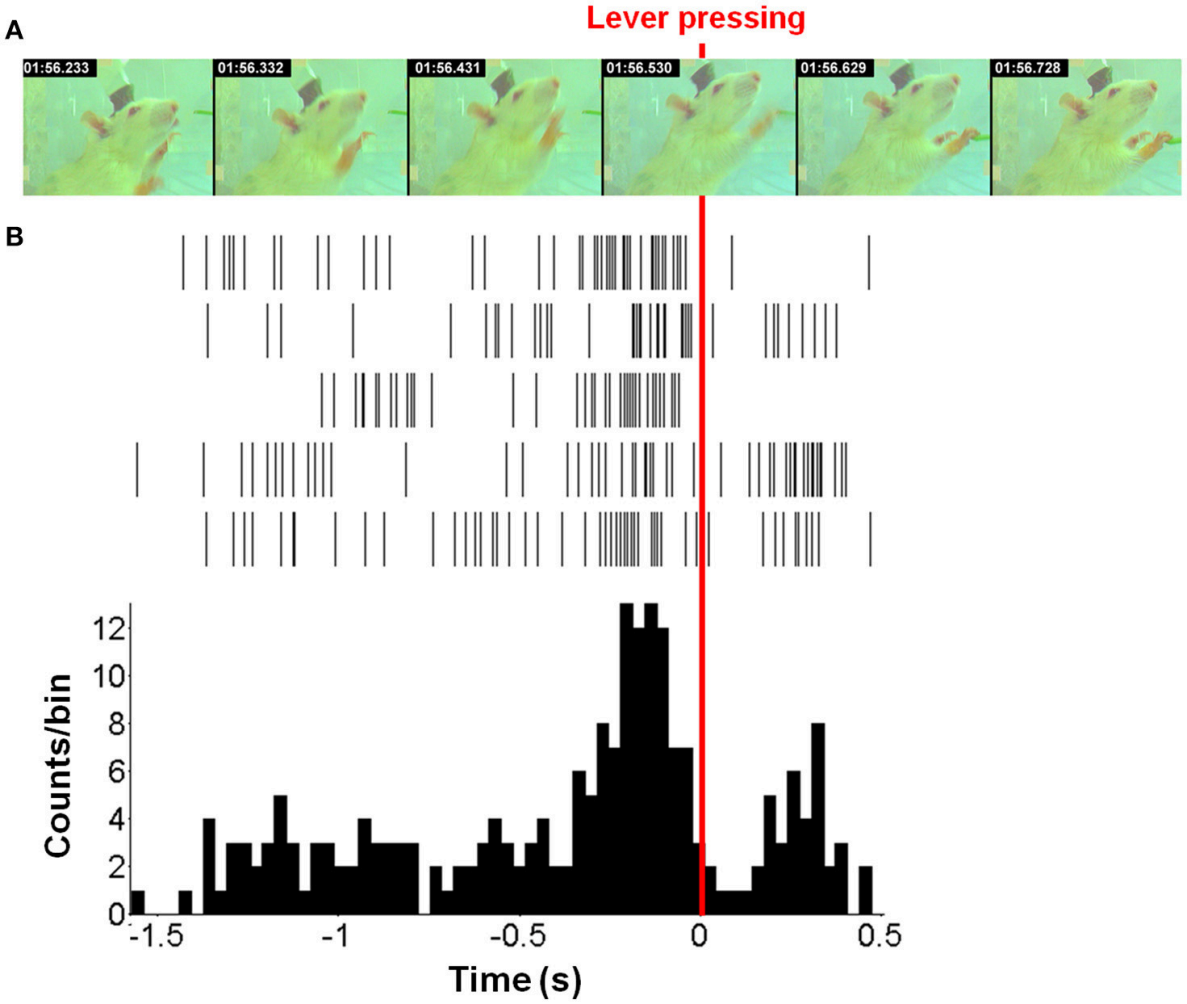
**One example of forelimb movement over time. (A)** The movement video of forelimb movement. **(B)** The neuronal activities were recorded from five neurons during one movement displayed as spike trains and the neuronal activity histogram (a bin size of 33 ms). The red line indicated the moment when the rat presses down the lever with the right-forelimb. Moreover, our results showed that neuronal firing rates highly correlated with forelimb movement; >71% (41/57) neurons exhibited specific firing changes during movement used to discriminate directional pairs.

The task timeline in Figure 2 presents the sequential images of the lever-pressing (Figure 2A) and the corresponding spike trains (Figure 2B). The spike trains were acquired from five neurons related to right forelimb movement and were represented as the neuronal activity histogram with 33 ms time bins. The neuronal activities distinctly increased approximately 0.4 s before lever-pressing (Figure 2B), corresponding to the second image at 01:56.332 s (Figure 2A). The maximum value in the histogram appears approximately 0.1 s before the lever-pressing. The neuronal activity has a substantial reduction after the rat completes the lever-pressing and then it re-strengthens gradually because of the redundant movement off of the lever.

### Effects of different time-lags and temporal orders

**Figure 3:**
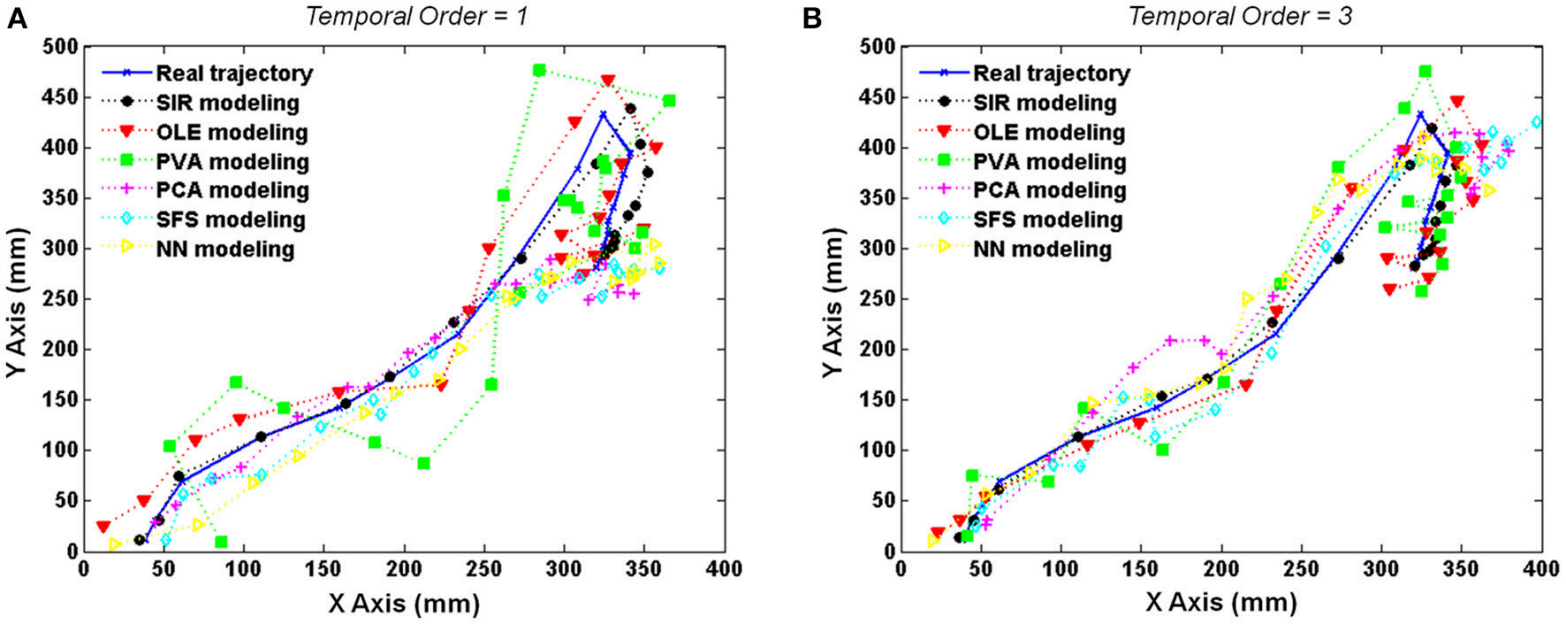
**Reconstructed trajectories of the test trial with the use of delayed activity with four time-lags (132 ms)**. The actual trajectory (blue solid line) and the trajectories predicted by SIR (black dashed line), OLE (red dashed line), PVA (green dashed line), PCA (magenta dashed line), SFS (cyan dashed line), and NN (yellow dashed line) are shown for an example trial using **(A)** one- and **(B)** three- temporal orders. The trajectory reconstructed by SIR is more accurate than the other methods.

Results of a particular test trial (Rat 106, 29 units, and 52th trial) were shown in Figure 3 where the trajectories were reconstructed by six decoding methods with delay activity of four time-lags. The actual trajectory (blue solid line) was compared with the decoded trajectories by SIR (black dashed line), OLE (red dashed line), PVA (green dashed line), PCA (magenta dashed line), SFS (cyan dashed line), and NN (yellow dashed line) in Figure 3. Furthermore, the results of one and three temporal orders were conducted to demonstrate the advantage of SIR with the requisite amount of input data as shown in Figures 3A,B, respectively. In the one temporal order experiment, the trajectories reconstructed by PVA and OLE obviously deviated from the actual trajectory more than that reconstructed by SIR. PCA and SFS, which perform feature selection, could achieve accurate prediction in the previous time steps but did not predict the latter time steps well. Similarly, a NN, which has learning ability for nonlinear regression, could predict the trajectory well in the first few time steps, but it did not have robust prediction performance because of the random initialization of weights that leads to prediction error. As the temporal order increased to three, all methods had more accurate prediction compared to those of one temporal order. Overall, SIR shows the best performance among the other methods, especially when the decoding methods used less neuronal activity information.

**Figure 4:**
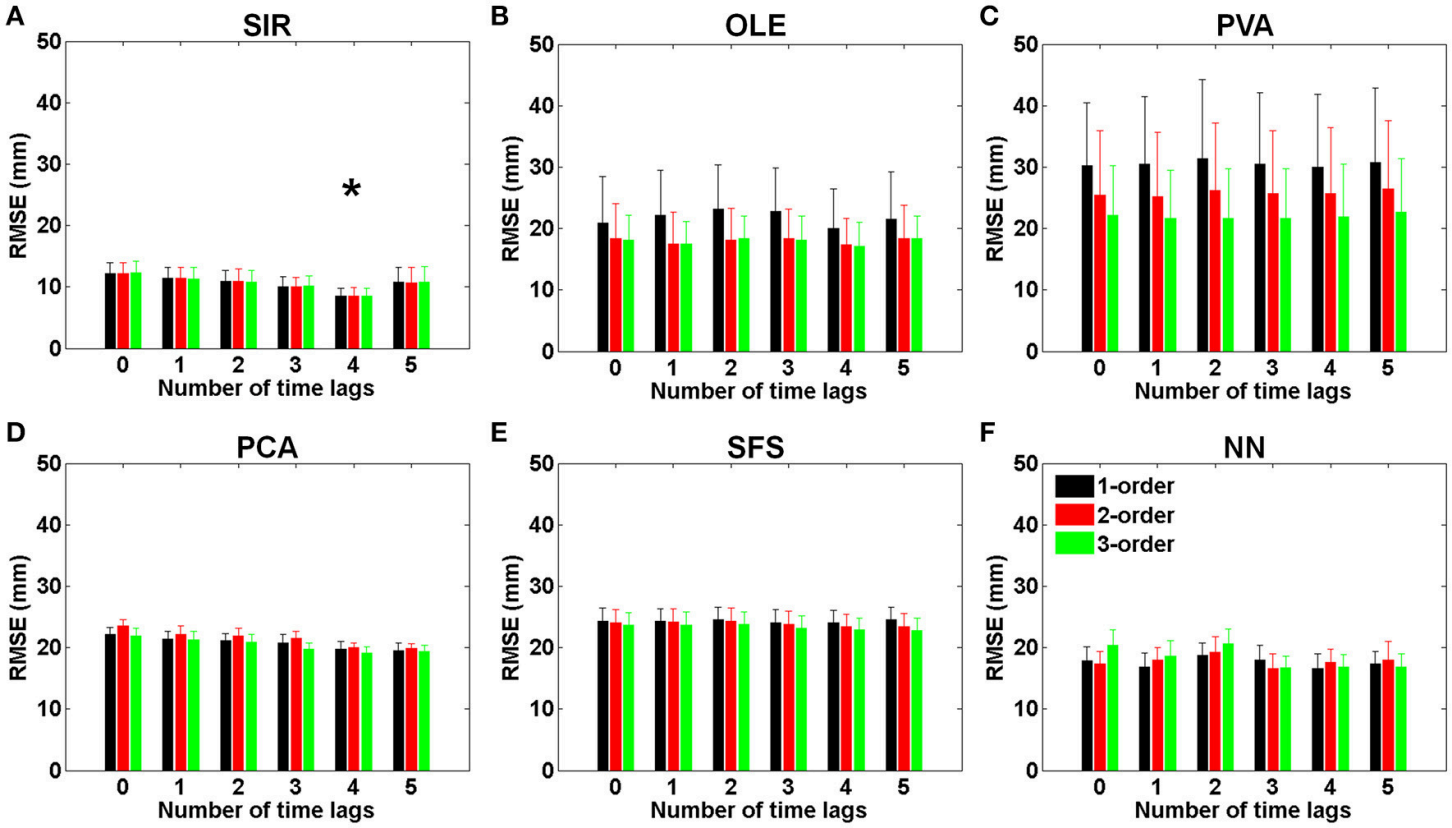
**RMSEs of (A)** SIR, **(B)** OLE, **(C)** PVA, **(D)** PCA, **(E)** SFS, and **(F)** NN decoding methods plotted with various time-lags (33 ms/lag) and temporal orders. The error bars denote standard error of the mean (Mean ± SEM). The results showed that SIR is superior to the other methods for trajectory reconstruction. SIR is unaffected by temporal orders, and the best performance was achieved with four time-lags (132 ms). The symbol ^*^ indicates significant different means with *P* < 0.002 and analyzed by Bonferroni correction for multiple comparisons, *N* = 145. Mean ± SEM%.

Figure 4 presents the effects of various time-lags and temporal orders in each method. Figure 4A shows the results of SIR where the smallest RMSE (8.47 ± 1.32 mm) was obtained by using four time-lags (132 ms) and one temporal order. It can be seen that SIR with four time-lags could achieve a significantly smaller RMSE than those with various time-lags [*F*_(5, 54)_ = 4.22, ^*^P < 0.002 with Two-way ANOVA with Bonferroni correction, *N* = 145]. Furthermore, the RMSE of SIR had no conspicuous variations among the three different temporal orders. As shown in Figure 4B, OLE achieved the smallest RMSE (17.22 ± 3.80 mm) by using four time-lags and three temporal orders. However, there was no significant enhancement of the prediction performance using OLE decoding with different time-lags and temporal orders. Figure 4C shows the results of PVA where the RMSE decreased as the number of temporal orders increases. PVA resulted in an average RMSE of 21.76 ± 8.11 mm when using one time-lag and three temporal orders. Figures 4D,E showed the results of PCA and SFS, respectively, where the features were selected via these two algorithms. PCA achieved a decreasing RMSE as the number of time-lags increased and obtained the smallest RMSE (19.13 ± 0.75 mm) when using four time-lags and three temporal orders. The results of SFS did not present a decreasing RMSE as the number of time-lags increased. SFS achieved the smallest RMSE (22.75 ± 2.01 mm) when using five time-lags and three temporal orders. Figure 4F shows the results of NN where the smallest RMSEs (16.75 ± 2.02 mm) were achieved by using three time-lags and two temporal orders. The RMSEs of NN did not present a regular trend as the number of time-lags increased. The forelimb movement predictions using OLE, PVA, PCA, SFS, and NN were not affected by either time-lag or temporal order. No significant interaction of time-lag and temporal order was found in the decoding methods of OLE, PVA, PCA, SFS, and NN in comparison to SIR. These results indicated that SIR outperformed other methods for trajectory prediction.

## Discussion

The main finding of this study is that a rat's forelimb movement could be successfully predicted and reconstructed using relatively few motor cortical neurons. In comparison with competing neural decoding algorithms including PVA, OLE, PCA, SFS, and NN, SIR presented an extremely superior RMSE in distance deviation between the reconstructed and real forelimb movement trajectories.

Previous studies indicated that neuronal activity discharged before the onset of the desired movement, such as the motor preparation period, and encoded behaviors (Chapin et al., 1999; Churchland et al., 2006). The kinematic parameters therefore were decoded and reconstructed with high accuracy using the neuronal activity before the onset of the movement. Hence, in this study, SIR and the competing algorithms decoded the neuronal activities during the motor preparation period for reconstruction of introduced upcoming lever-pressing. The results showed that SIR, OLE, and PCA achieved optimal efficiency when using the neuronal activities that led to the onset of forelimb movement for 132 ms. PVA, SFS and NN each achieved optimal efficiency by using the neuronal activities that led to the onset of forelimb movement for 33, 165, and 99 ms, respectively. In Figure 2B, the peak of the spike train occurred at four time-lags, prior to the onset of lever-pressing. The neuronal firing rate then declined for 0.2 s as a result of the completion of motor command transmission. Because the rat performed an unexpected forelimb swing, the neuronal firing rate increased again approximately 0.2 s after to the onset of lever-pressing.

The performance of cortical neural decoding hinged on the exploited information in chronically-recorded neuronal activities. Previous studies using PVA (Schwartz, 1994) and OLE (Salinas and Abbott, 1994) show that cortical neurons with known motor associations were chronically sampled and as many as possible were recorded. Because of the lack of a precise technique to target the modulated neurons that largely contributed to goal-directed behavior, PVA and OLE summed the weighted vectors across all neurons, performing a neuronal vote, to predict the kinematic parameters (Salinas and Abbott, 1994; Schwartz, 1994). A large number of electrodes and sample neurons (usually up to hundreds) was required for reconstruction of kinematic parameters with a high degree of accuracy (Chapin et al., 1999; Wessberg et al., 2000; Serruya et al., 2002; Taylor et al., 2002). However, the neuronal activity was not as stable from day to day (Sadtler et al., 2014). PVA and OLE may be affected by neuron's stability since they extract movement information from the selected cortical population.

In this study, we recorded only tens of neurons from rat M1 where the amount of recorded neurons was insufficient for PVA and OLE, which usually require hundreds of neurons to provide a robust neural decoding process (Takeda and Funahashi, 2004; Wahnoun et al., 2006). Compared to PVA and OLE, SIR can effectively achieve nonlinear regression from a small number of inputs (Li, 1991). SIR adopted a sliced regression framework with a sorting procedure to divide the neuronal dataset into several slices according to the sorted output variable value. Each slice contained neurons with a similar contribution to the introduced lever-pressing and was then modified by a weight. Slices containing neurons with tiny or even a null contribution to the lever-pressing may gain zero weight and can be removed from the decoding model. Multiplied by a proper weight according to the weighted PCA, a slice containing a few neurons with a high contribution presented a comparable influence on the prediction and reconstruction of the introduced lever-pressing to the neuronal vote from hundreds of neurons. Hence, SIR is able to perform forelimb prediction through a small number of neurons. On the other hand, dimensionality reduction technique factor analysis is usually adopted to describe population activity using low-dimensional set of factors and highlight feature of interest in data from a large number of recorded neurons (Sadtler et al., 2014). Although SIR could perform dimensionality reduction through weighted PCA, it preserved all recorded neurons and assigned weights to the slices according to their contribution. It learned forelimb movement prediction from whole neuronal activities regardless of neuron's stability across days. Thus, SIR was robust to uncertain variation of movements and neuronal activities across days due to the success of inverse regression and effective dimension reduction. Furthermore, using this SIR, the size of the neural decoding model topology was significantly reduced, burdened with data storage and reduced computational loading, indicating that efficiency in neural decoding in comparison to PVA and OLE was attainable. Compared to PCA and SFS, which perform feature selection and dimensional reduction, PCA could further project the data onto another space, which could lead to a better reconstruction than SFS. However, SIR outperformed PCA and SFS because SIR clusters data into each slice according to the output values. NN performed better prediction than PCA and SFS because of its nonlinearity and learning ability. Nevertheless, NN did not result in a robust reconstruction because of the mechanism of random initialization and the existence of many local optima. These comparisons indicate that the neural decoding based on SIR with one temporal order presents a smaller RMSE in reconstructing limb movement than those based on PVA or OLE with three temporal orders and those based on feature selection and learning ability. It indicates that SIR can be a more suitable solution than the commonly used linear progression methods using the neuronal ensemble inputs to predict and reconstruct the introduced limb movement.

## Conclusions

Neural decoding models that require hundreds of input variables, such as PVA and OLE, not only require considerable computation but also have detrimental effects in the decoding process because of errors in assigning neuronal spikes or non-stationary noise, especially for non-adaptive models. Reducing the neurons that may cause model over-fitting emerges as a significant neural decoding issue. However, with the help of the proposed approached based on SIR, researchers can predict and reconstruct the limb movement of interest with high accuracy using only tens of neurons in a single setting. Furthermore, SIR outperformed other feature selection methods, such as PCA and SFS because of its clustering ability. SIR further achieved more robust performance than NN because there is no random initialization and local optimization problems in SIR. By indexing the contribution of multiple cortical areas with different sizes, it has become feasible to ascertain the importance of selected areas for the motor commands. This will provide valuable insights for follow-up studies in the future.

## Ethics statement

The animal use protocol listed below has been reviewed and approved by the Institutional Animal Care and Use Committee (IACUC) at the National Yang Ming University. Protocol Title: Peripheral Nerve Prostheses: A Paradigm Shift in Restoring Dexterous Limb Function. IACUC Approval No: 1050622. Period of Protocol: Valid From: 08/01/2016. To: 07/31/2020. There is no endangered animal species involved in this study.

## Author contributions

SY, HYL, and YC designed the project, organized the entire research. SL, LL conceived the experiments. CW, PC, TP, HCL, and WH conducted the experiments. HC, HHSL, and YL analyzed the results. SY, HYL, and YC wrote the manuscript. All authors discussed the results and reviewed on the manuscript.

### Conflict of interest statement

The authors declare that the research was conducted in the absence of any commercial or financial relationships that could be construed as a potential conflict of interest.
