# Supplementary material for: A Sliced Inverse Regression (SIR) Decoding the Forelimb Movement from Neuronal Spikes in the Rat Motor Cortex
Source: Front Neurosci. 2016 Dec 9;10:556. doi: 10.3389/fnins.2016.00556 (PMC5145870; doi:10.3389/fnins.2016.00556)
Supplement: Supplementary file 1 [file DataSheet1.DOCX]

Supplementary Material

A sliced inverse regression (SIR) decoding the forelimb movement from neuronal spikes in the rat motor cortex

Shih-Hung Yang^1,+^, You-Yin Chen^2,+^, Sheng-Huang Lin^3,4^, Lun-De Liao^5,6^, Horng-Shing Lu^7^, Ching-Fu Wang^2^, Po-Chuan Chen^2^, Yu-Chun Lo^8^, Thanh Dat Phan^1^, Hui-Ching Lin^9^, Hsin-Yi Lai^10,*^and Wei-Chen Huang^11^

^1^ Department of Mechanical and Computer Aided Engineering, Feng Chia University, No. 100, Wenhwa Rd., Taichung 407, Taiwan, ROC

^2^ Department of Biomedical Engineering, National Yang Ming University, No.155, Sec.2, Linong St., Taipei 112, Taiwan, ROC

^3^ Institute of Biomedical Engineering, College of Medicine, National Taiwan University, No.1, Sec. 1, Jen-Ai Rd., Taipei 100, Taiwan, ROC

^4^ Department of Neurology, Tzu Chi General Hospital, Tzu Chi University, No. 707, Sec. 3, Chung Yang Rd., Hualien 970, Taiwan, ROC

^5^ Institute of Biomedical Engineering and Nanomedicine, National Health Research Institutes, 35 Keyan Rd., Zhunan Town, Miaoli County 350, Taiwan, ROC

^6^ Singapore Institute for Neurotechnology (SINAPSE), National University of Singapore, 28 Medical Drive, #05-COR, 117456, Singapore.

^7^ Institute of Statistics, National Chiao Tung University, Assembly Building I, 1001 Ta Hsueh Road, Hsinchu 300, Taiwan, ROC

^8^ The Ph.D. Program for Neural Regenerative Medicine, Taipei Medical University, No. 250 Wu-Hsing St., Taipei 110,Taiwan, ROC

^9^ Department and Institute of Physiology, School of Medicine, National Yang Ming University, Taipei, Taiwan 112, ROC

^10^ Interdisciplinary Institute of Neuroscience and Technology, Qiushi Academy for Advanced Studies, Zhejiang University, 268 Kaixuan Road, Hangzhou, Zhejiang, 310027 China.

^11^ Department of Materials Science and Engineering, Carnegie Mellon University, No.5000 Forbes Avenue, Wean Hall 3325, Pittsburgh, PA 15213, USA

^+^ Shih-Hung Yang and You-Yin Chen contributed equally to this work.

^*^Correspondence should be addressed the following:

Dr. Hsin-Yi Lai, Interdisciplinary Institute of Neuroscience and Technology, Qiushi Academy for Advanced Studies, Zhejiang University, No.268, Kaixuan Rd., Hangzhou, Zhejiang310029, China

Email: laihy@zju.edu.cn

*Note 1. Forelimb Trajectory and Neuronal Signals*

To record forelimb movement, each rat was tagged with a colorful ring on its right forelimb as a tracking marker before each recording session. A charge-coupled device (CCD) camera (DFK 21F04, Imaging Source, Bremen, Germany) was mounted perpendicular to the lever direction at a fixed distance (25 cm) from the lab-designed Plexiglas testing box. The rat was unchained in the Plexiglas testing box during the experiments. The lever-pressing forelimb movement for the water reward was captured at 30 Hz in 640 × 480 RGB format by the CCD camera and was processed by a Plexon CinePlex video tracking system (CinePlex, Plexon Inc., Dallas, TX, USA). This study intended to predict forelimb movement based on neural activities in the M1 cortex when the rat presses a lever to obtain a water reward. Because the height of the lever is higher than the rat, the rat needs to perform bipedal standing to press the lever with its forelimb. The recorded trajectory of the colorful ring might consist of movements of both the forelimb and the whole body. This study assumes that the whole body performs a very small movement as the rat completes bipedal standing. Therefore, the approximate forelimb movement was recorded from a small window in the sagittal view where the forelimb appeared in the window as the rat completes bipedal standing; this view was expected to eliminate the effect of the whole body movement from the recorded trajectory. Thus, a sagittal view with a 120 × 90 mm window was carefully selected to obtain the approximate forelimb movement where the colorful ring would appear in the window when the rat presses the lever with its forelimb during bipedal standing.

Additionally, the neuronal activity data in the M1 cortex were collected through a 16-channel stainless microwire electrode array and were recorded by a Multi-channel Acquisition Processor (MAP, Plexon Inc., Dallas, TX, USA). The recorded neuronal signals were then transmitted from the head stage to an amplifier through a band-pass filter (spike preamp filter: 0.3–5 kHz; gain: 15,000–20,000) with a 40 kHz sampling rate. To capture the instant lever-pressing behavior, a positive 2.2 voltage pulse was generated through the MAP system when the rat started to press the lever. In addition, spikes (i.e., action potentials) from each electrode were detected with the amplitude threshold and were classified with template-sorting algorithms using commercial software (SortClient v2.5.2, Plexon Inc., Dallas, TX, USA); single units were then identified and isolated from the recorded neuronal signals. Here, the neuronal signal was segmented into time bins with a length of 33 ms, which is equal to the temporal resolution of the video tracking system; then, the firing rate was calculated by counting the spikes within each time bin.
